# A case study evaluation of implementation of a care pathway to support normal birth in one English birth centre: anticipated benefits and unintended consequences

**DOI:** 10.1186/1471-2393-9-47

**Published:** 2009-10-05

**Authors:** Debra E Bick, Jo Rycroft-Malone, Marina Fontenla

**Affiliations:** 1 Division of Health and Social Care Research, King's College London, James Clerk Maxwell Building, 57 Waterloo Road, London, UK; 2 Centre for Health Related Research, School for Healthcare Sciences, University of Wales, Bangor, UK; 3 RCN Research Institute, University of Warwick, Warwick, UK

## Abstract

**Background:**

The policy drive for the UK National Health Service (NHS) has focused on the need for high quality services informed by evidence of best practice. The introduction of care pathways and protocols to standardise care and support implementation of evidence into practice has taken place across the NHS with limited evaluation of their impact. A multi-site case study evaluation was undertaken to assess the impact of use of care pathways and protocols on clinicians, service users and service delivery. One of the five sites was a midwifery-led Birth Centre, where an adapted version of the All Wales Clinical Pathway for Normal Birth had been implemented.

**Methods:**

The overarching framework was realistic evaluation. A case study design enabled the capture of data on use of the pathway in the clinical setting, use of multiple methods of data collection and opportunity to study and understand the experiences of clinicians and service users whose care was informed by the pathway. Women attending the Birth Centre were recruited at their 36 week antenatal visit. Episodes of care during labour were observed, following which the woman and the midwife who cared for her were interviewed about use of the pathway. Interviews were also held with other key stakeholders from the study site. Qualitative data were content analysed.

**Results:**

Observations were undertaken of four women during labour. Eighteen interviews were conducted with clinicians and women, including the women whose care was observed and the midwives who cared for them, senior midwifery managers and obstetricians. The implementation of the pathway resulted in a number of anticipated benefits, including increased midwifery confidence in skills to support normal birth and promotion of team working. There were also unintended consequences, including concerns about a lack of documentation of labour care and negative impact on working relationships with obstetric and other midwifery colleagues. Women were unaware their care was informed by a care pathway.

**Conclusion:**

Care pathways are complex interventions which generate a number of consequences for practice. Those considering introduction of pathways need to ensure all relevant stakeholders are engaged with this and develop robust evaluation strategies to accompany implementation.

## Background

During the last decade, the policy drive for the UK National Health Service (NHS) has focused on the provision of a modernised, high quality service informed by 'best practice' and evidence of 'what works'. In England, the aim of health reform is *"to develop a patient-led NHS that uses available resources as effectively and fairly as possible to promote health, reduce health inequalities and deliver the best and safest healthcare*" [1]. There has been a proliferation of agencies responsible for synthesising and disseminating evidence of clinical and cost effective interventions for NHS care, for example the National Institute for Health and Clinical Effectiveness (NICE), and for monitoring the safety and quality of care including the Care Quality Commission and the Health Protection Agency. Evidence based guidance published by NICE and evidence based standards presented within National Service Frameworks (NSF) for priority health areas, including Children, Young People and the Maternity Services [2] have explicitly referred to the use of care pathways and protocols to standardise care and sustain the implementation of evidence in practice as well as stimulate more proactive engagement of service users in decisions about their care.

Alongside the shift in the policy context, the roles of health professionals in the UK have been changing to reflect the need for a more responsive and flexible workforce, with recognition that some traditional professional boundaries will need revision if quality of care and service user engagement in healthcare decisions are to improve [1]. This has affected all aspects of service delivery, including maternity care. The introduction of the Working Time Directive (WTD) which reduced the number of hours junior doctors work, has also contributed to the need to revise ways of working within the maternity services with more emphasis placed on the roles and responsibilities of midwives. In addition to utilizing skills more appropriately, revisions to midwifery working practices are viewed as important to increase job satisfaction [3]. Recent strategy for the maternity services [3] outlined how women and their partners should be at the centre of maternity service provision and how relevant stakeholders, including women and midwives can use the health reform agenda to shape care to meet the needs of individual women and their families.

The current policy agenda, with an emphasis on timely and effective care informed by evidence of quality and safety and provided by the most appropriate health professional is ambitious. Although the delivery and standardisation of care has been promoted through the use of protocols, there has been minimal systematic evaluation of the impact of these on practice or patient care. Protocol-based care remains a poorly understood term and can potentially encompass the use of care pathways, integrated care pathways, guidelines, algorithms and checklists [4,5]. Studies which have examined standardised care practices in nursing found protocol use legitimised nursing knowledge [6], however in contrast medical staff were unlikely to use standardised approaches, as they would rely on previous experience and information from education and journals [7,8]. Although there is a large literature base with respect to protocol based care to inform a wide spectrum of nursing and medical interventions [9], there is limited literature of the development or impact of use of protocols in midwifery. A large cluster randomised controlled trial of a new model of protocol based midwifery-led community based postnatal care, which included a 'package' of interventions and midwifery contact over an extended period of time was associated with enhanced maternal mental health outcomes at 4 and 12 months after birth [10,11]. Evaluation of midwives views of postnatal care showed that the intervention midwives were more positive about their role, felt the new model made better use of their skills and time and was more appropriate to meet the needs of the women [11]. A survey of use of a protocol to inform smoking cessation among representatives from all of the then 466 Dutch midwifery practices found use was more likely to be influenced by personal awareness and motivational factors rather than organisational factors [12]. If protocol based care is to make the difference to health outcomes and health professional roles as anticipated in policy recommendations, questions remain about how approaches to standardise care are used in practice and what their impact is on midwives, nurses and other health professionals' roles, service delivery and multi-disciplinary working. Concerns have been raised that pathways to standardise care are complex documents, as use combines a framework for decision making within an individual patient record, with potential tensions arising between providing standardised care and offering individualised care. The consequences of the subsequent reduction in the use of documentation associated with the use of pathways are as yet unknown [5].

This paper reports findings from a case study evaluation of the impact of the use of a care pathway to support normal labour in one NHS Birth Centre on midwifery practice and women's views of their care. Data were collected as part of a larger study of the use and impact of protocols in clinical settings [13,14]. The study was methodologically underpinned by realistic evaluation [15] and as the evidence base to support protocol based care was so variable, aimed to explore and explain use by studying standardised approaches in the reality of the clinical setting.

### Design

Realistic evaluation was the over-arching framework for the study [15], as it acknowledges the importance of context to an understanding of why interventions and strategies work, for whom, how and in what circumstances. The explanatory proposition is that programmes work (have successful *outcomes - O*) only in so far as they introduce appropriate ideas and opportunities (*mechanisms - M*) to groups in the appropriate social and cultural conditions (*contexts - C*). Realistic evaluation enables the relationship between *mechanisms, outcomes *and *context *to be determined and the relationships between them to be explored. For this study we were interested in finding out how the normal birth pathway (*mechanism*) impacted on midwives, women and service delivery (*outcomes*) within the service delivery setting (*context*).

A case study design was used to capture data on the use of protocols within the clinical site [16,17] which was methodologically complementary to Pawson and Tilley's framework [15]. This approach enabled a focus on protocol-based care within its real life context, the utilisation of multiple methods and an opportunity to study and understand the experiences of different stakeholder groups [16]. Yin's definition of a case study was used [16]; a 'case' was the clinical setting (the Birth Centre) and the 'embedded unit' of the case was the use of the care pathway to support normal labour.

### Setting

The study site was purposively sampled using the following criteria:

- active engagement in the use of protocol-based care

- the opportunity to study midwife-led care

- feasible travelling distance to the researcher's place of work

- willingness to participate

In the first instance a short list was developed of possible sites fitting these criteria. Senior managers were then approached to ascertain whether they would in principle be willing to participate. Managers from the maternity unit where the Birth Centre was based expressed an interest and willingness to be involved as six months prior to commencing the study, an adapted version of the All Wales Clinical Pathway for Normal Birth [AWP, 18] had been implemented and they were interested in evaluating its impact.

The AWP was introduced into Welsh maternity units during 2002 - 2004 as a key strategic response to concerns over rising caesarean section rates. It forms a core part of Welsh maternity policy aimed at the promotion of normal birth and subsequent reduction in interventions in women who had low risk pregnancies [18,19]. All midwives in the study Birth Centre were using the pathway, which was the only record of a woman's care following her admission to the Birth Centre, through her labour and birth until her transfer to the postnatal room. If a woman was assessed for signs of early labour, the relevant section of the pathway was commenced, even if she was later discharged home if labour was not established.

Following piloting at the Birth Centre, which included introductory sessions for the midwives on use of the pathway by the Lead Midwife for Normal Birth, it had been adapted to produce a two part, rather than a three-part document. The main change was the removal of the original Part 1 of the pathway which was a section to record advice following telephone consultations. Assessments during the latent phase of labour were also to be undertaken every hour and not every 30 minutes as recommended in the original pathway. It was the only pathway used to inform normal labour in the hospital at the time and was only in use on the Birth Centre. Other protocols in use at the hospital, but not the Birth Centre which only accepted low risk women, included third stage management, antenatal complications and antenatal admissions.

The Birth Centre opened in 2005 and was based on one ward of a large maternity unit in the South of England. The same NHS Trust also had a smaller midwifery-led unit on a separate site. The Birth Centre was staffed by a core team of midwives, managed by the Lead Midwife for Normal Birth, assisted by maternity support workers and housekeeping staff. Most of the midwives who elected to work on the Birth Centre had previously worked in the same maternity unit on the main delivery suite or in the community. Student midwives were rotated to the unit and there was no routine obstetric involvement. At the time of conducting the research, Birth Centre staff cared for between 50-70 women a month, with plans to increase capacity. There were four birth rooms each with an en-suite bathroom, one postnatal area with six beds, an antenatal assessment room, a lounge for women and their partners, bathroom facilities for women and their partners, a kitchen and a midwife office.

There were twice weekly tours of the Centre for prospective parents to provide an opportunity to ask about care and for the midwives to explain the philosophy of the Centre, which included that care was non-interventionist, the woman and her birth-partner were actively engaged in decisions about care and the woman would be encouraged to be as mobile as possible during her labour. In addition to options for pain relief which could be offered and managed by the midwives (entonox, birthing pool and IM pethidine), midwives at the Centre also offered reflexology. Weekly antenatal clinics were held for women booked at the Centre, a recent initiative when the study commenced. At the time of undertaking the study, the transfer rate to the main labour ward was 24%, the majority of transfers being for women who requested epidural analgesia or because the midwives had identified meconium stained liquor.

## Methods

Various sources of evidence to understand the use of the labour and birth pathway were used, including non-participant observations which were guided broadly by Spradley's nine dimensions of observation, including space, actors, activities, objects, acts, events, time, goals and feelings [20], semi-structured interviews with women, midwives and key stakeholders guided by interview schedules developed by the study team [9] and the labour pathway documentation. Field notes following each period of observation were written by the researcher to provide details of particular events during the observation period. Non-participant observation of a sample of pathway-based care interventions during labour took place on the Birth Centre after a period of general non-participant observation to raise awareness of occasions that might be observation opportunities. This elicited that a period of observation of women during labour (the second part of the pathway) would provide the most valuable insight into the use of the care pathway in practice, rather than the first part of the pathway (care on admission to the Centre).

Following a completed observation, interviews were undertaken by the first author (DB) with the woman and with her midwife, the content of which was guided by particular incidents or issues which had arisen during the period of observation. The researchers were keen to elicit what women thought about the quality of the care they received and if they were actively engaged in decision making. The aim of the interviews with the midwives who had been observed was to explore their role in the application of the care pathway, their perceptions of the benefits and drawbacks, facilitators and barriers to the use of the pathway and the potential for further developing and improving this form of care delivery. Questions about the impact of the pathway on their professional identity, responsibility and autonomy were also included. As time of onset of labour was unpredictable and a period of observation would have to take place over a 24 hour period, it was unlikely that all women recruited would be observed and this was explained to women when consent to take part was sought. A decision was taken by the research team that interviews with women who had not been observed and the midwives who had cared for them could also be conducted. These interviews would provide valuable data on midwives' and women's experiences of the pathway, the only difference being that the researcher perspective of care would be missing. Key stakeholder interviews were held with senior midwifery managers, midwives working on the Birth Centre who were not observed and obstetricians to explore their views of the care pathway, its use in practice and influences on use. All interviews were audio-recorded and transcribed in full. Data were collected during 32 days between March 2006 and January 2007. Observations took place at different times over a 24 hour period with each period of observation lasting for between two - three hours to enable issues relevant to the use of a care pathway on a woman's care and her progress in labour to be noted.

### Data analysis

Qualitative data were content analysed following the approach described by Huberman and Miles [21] and Yin [16] which follows a process whereby data are 'made sense' of through coding, developing themes and patterns, and relationships. An inductive process was used to break data into codes within each data set (interviews, observations and documents), with codes then developed into themes. Data analysis was undertaken by three members of the study team (DB, MF, JRM), which enabled the challenging and or confirmation of coding, theme development and interpretation. Data analysis was managed in QSR Nudist (v5). Codes were used to anonymize the interviewees; 'SO' indicates an interview with a key stakeholder, 'MO' with a midwife from the Birth Centre and 'WO' a woman who had recently given birth.

### Ethics

Multi-site Research Ethics Committee approval was obtained for the study. Site specific approval was also required for the NHS Trust where the Birth Centre was based. Women attending the Birth Centre were approached at their 36 week antenatal consultation by DB and MF and offered an information leaflet about the study. Those who wished to give their consent at the time could do so, while other women preferred to have time to think about taking part and provide a response on whether they wished to take part at their next antenatal appointment.

## Results

### Recruitment

Twenty-six women were recruited from the Birth Centre. All were around 36 weeks gestation when recruited. A total of 18 interviews were conducted which included four women observed during labour (three nulliparous and one multiparous woman) and interviewed post-observation, and interviews with each of their attending midwives. Two women who were not observed were also interviewed (one nulliparous and one multiparous). Interviews with women were conducted following hospital discharge, within 4 weeks of the birth. Eight interviews were held with key stakeholders, which included two obstetricians, three senior midwives (holding managerial responsibility for different parts of the maternity service) and three midwives who had worked with the Birth Centre manager to introduce the pathway into practice.

### Identified Themes

#### Development and introduction of the pathway - 'a midwifery thing'

The introduction of the pathway was viewed as key to support the philosophy of care and midwifery skills in normal birth which were promoted within the Birth Centre. There was a strong feeling that the pathway had been introduced into an area very receptive to its use, with the midwifery staff fully supportive of its introduction. However, on reflection, senior midwives interviewed as key stakeholders felt that the approach to introduction into the Centre and the organisation as a whole could have been handled differently. There was no involvement of the obstetricians or midwives working on the main labour ward in the adaptation or implementation of the pathway, which the obstetricians initially viewed as a 'midwifery' thing. As a result there were issues identified with respect to the impact on relationships with obstetric and midwifery colleagues on the labour ward, with one midwife reflecting on whether the introduction of the pathway should have been more inclusive with respect to other members of the midwifery workforce:

*'Introduction of the pathway was bound to be successful because we've used it in a very, what's the word, receptive, you know, motivated area and think that although we did talk to the whole staff about it, I think going back again there should be more inclusion of the rest of the organisation.... I think generally we should have made more general awareness across the rest of the midwifery workforce' *(S02)

There was also some reflection that the introduction of the pathway should have followed a more formal process prior to its introduction, as one of the senior midwifery managers raised:

*'I think in some ways its (introduction) needed to be more formal. We did discuss it at the labour ward or Directorate Clinical Governance meetings but it was discussed once it was put in place. This is what we're going to do, this is what we've got. The obstetricians said that's fine at that point, we don't have to use it anywhere so we don't need any input' *(S06).

### Impact on midwifery role

All midwives on the Birth Centre used the pathway to inform care of a woman during her labour and birth as it was the only documentation in use. During the observation periods, it was clear that the pathway remained outside of the birth room and was kept at the midwives desk in the main area of the Centre. When the midwives were asked how they used the pathway, responses indicated that use supported rather than informed their practice. The pathway was not viewed as a substitute for the midwife's clinical judgement, it was there to support their decisions.

*'I think it's still a matter of the clinical experience of the midwife - it gives prompts, for example, if labour isn't progressing, but as a midwife you should not be solely relying on the pathway. You have to use your clinical judgement at all times. What it does is support your actions for women in normal labour, if you like, decisions about what to do next, but to be honest this is what a good midwife should be doing anyway *(S05)

The midwives had both negative and positive views about the introduction of the pathway and impact on their practice. One area which was frequently reported was difficulty in adjusting to the minimal level of recording that the pathway required. Following the introduction of the pathway, it was apparent that this was the most problematic aspect as illustrated in the following quote and it was mentioned by several of the midwives:

*'probably ... one of the hardest things ...in terms of stepping back and not writing' *(S02)

From interviews with key stakeholders, the reduction in documentation followed a 'learning curve', with some of the midwives finding it difficult to adapt to use of a pathway which did not require substantive comments with respect to the progress of a woman's labour:

*'..they struggled with not writing. It wasn't because they didn't think the pathway was good, they couldn't get their heads around not documenting. I would read notes two or three weeks of opening (the Birth Centre) and they would still be handling notes along with the pathway' *(S03)

There was also concern expressed about what happened to the woman if she had to be transferred to the labour ward, where the pathway was not in use. The Birth Centre midwife had to ensure other appropriate documentation was commenced to record what happened to the woman during and after her transfer:

*'I think the biggest thing is making sure that once women deviate from the pathway that they actually go onto other documentation, you know, that you document this and that this is properly documented in the notes etc appropriately. This is more of a concern, not so much what is on the actual protocol but what happens' *(S02)

### Autonomy

There were a couple of comments from one key stakeholder that some midwives who did not work on the Birth Centre felt that the introduction of a pathway took away a midwife's autonomy, as it told midwives 'what to do'. This perspective differed from that of the Birth Centre midwives who felt it reinforced their decisions about care:

*'...they find the whole pathway insulting because they feel they are being told what to do. They say it is not individualised because it says that you have to treat all women the same and not give them individualised care...they have got to see that you have to do more than just a, b and c. It's about laying it on the line and for you to use it as guidance. The midwives up here find it useful and say that 'if I have done x, y and z, I still have this to try before I do anything else'. Yes, I think up here they find it far more autonomous' *(S06)

With respect to midwives who had been qualified for longer (≥ 5 years), there was a view that some would have practised in a way which reflected the content of the pathway, regardless of whether it had been introduced or not:

*'Some of them would be doing it to some degree, and there are one or two (midwives) who spring to mind that I know, yes, would be practising this way regardless of where they are. But that comes with experience and that comes with confidence in what you're doing' *(S03)

For the newly qualified midwives who joined the Birth Centre team, using the pathway was viewed as a potential problem if they had to move later to the main labour ward as it encouraged them to work in a different way:

*'I think for sure the newly qualified midwives that we've had up here will be practising a different way from the labour ward for lots of reasons. Certain midwives working alongside them, the culture downstairs, the continued presence of obstetricians...sometimes it is about conforming rather than sticking your head above the parapet' *(S03)

### Confidence

For newly qualified midwives, use of the pathway was also a positive experience as it encouraged them to become more confident in their skills:

*'For a newly qualified midwife going into labour ward can be very frustrating in the sense that you've had all this training about normal birth and you don't see it as often as you would like to see it. The great thing is it encourages them to be more confident in their skills... for the newly qualified midwife, it has accentuated their training and given them confidence' *(S03)

The role of the Birth Centre manager who took the lead for the adaptation and implementation of the pathway in practice was also recognised as important to build team confidence and contribute to the successful implementation of the pathway. The pathway's impact on midwives' confidence was perceived to be due to the hard work of the manager and her role in preparing the midwives for the introduction of the pathway:

*'...xxx (manager) went to various presentations, looked at different units that use the All Wales pathway....xxx adapted that and is doing training sessions with the midwives, so they're trained in understanding what to do, how they need to document care. Xxx was very much hands on which has helped to build the confidence of the staff on the Birth Centre' *(S02)

As there had been issues with transfer of women from the Birth Centre to the main labour ward, discussions had taken place to rotate senior midwives from labour ward to the Birth Centre for a short allocation to develop their confidence in normal birth as well as to allay fears of working with the pathway which was the focus of their concerns:

*'Some of them have mixed fear and I think this will help with the fear because it is not only the pathway. It's the way we work up here, it's about water birth, it's about delivering different women, it's about not having a CTG. So it's not just about their fear of the pathway but this is what it's directed at because the pathway is tangible' *(S03)

### Litigation

The lack of written documentation within the pathway was a major concern for some key stakeholders, particularly if a woman's case was later referred to litigation because of an adverse obstetric outcome or complaint about her care. For the midwives on the Birth Centre, this meant they had to constantly defend their practice as the pathway did not require a record of every midwifery interaction or intervention; for the obstetricians there was a concern that the lack of documentation could indicate appropriate and timely care had not been given:

*'....we have a continual battle when we go to the perinatal Directorate Clinical Governance meetings with the obstetricians expecting a certain level of documentation. So everything can be very normal on the pathway, the woman is progressing as you expect her to and all of a sudden there is an issue. We had a case with a lady in the pool who had been fine. She was getting out of the pool and we just couldn't find the fetal heart. That needed an emergency transfer to the labour ward. It was then discussed at the perinatal meeting where it was clearly stated that the outcome was fine. However, the obstetricians found it difficult that there was no documentation leading up to that. We verbally said that this women showed no signs there was anything abnormal up until that point. They had the impression that maybe there were issues but we weren't documenting it' *(S03)

The same midwife also commented:

*'a fair proportion of my job is defending and standing strong that this is the right way forward for normal, low risk women... to try to get the obstetricians and senior midwives to understand that those decisions are regularly made on the labour ward when the cases are not managed as they should be, they highlight as an issue of confidence up here because the woman has come to the Birth Centre. They wouldn't dream of talking about a lack of confidence when it happens on the labour ward' *(S03)

From the obstetrician's perspective, it was felt that it was left to them to 'rescue' a woman's care, and that it was imperative that there was sufficient documentation of appropriate care if a case did get referred to litigation:

*'The only problem with all this, there is no documentation, there is no paper and if we had to go to a court of law there is nothing there, what do we do? There has to be some objective evidence that what you are doing you must be doing right' *(S04)

### Impact on team working

Use of the pathway was viewed as promoting good team working in the Birth Centre, with a particular emphasis on improving communication between midwifery staff:

*'I think the Birth Centre midwives find communication between each other easy. They all use the same paperwork, they can see any variations or anything that's happened. It does focus their minds on things that have happened that aren't within the pathway because it's documented' *(S03)

There was also a feeling that the introduction of the pathway had helped to retain midwives in practice as they had an opportunity to work on the Birth Centre:

*'I would say that we had a couple of midwives who were looking to leave and go elsewhere, but now they're not' *(S02)

The impact on working with colleagues on the labour ward had been most problematic:

*'You might say it's made things more difficult, but this may be something that changes when it becomes more accepted here. We do obviously communicate when we take a woman to labour ward, as we have to ensure we've handed over all relevant information to her care. In this case, the pathway would be one source of communication, not the whole thing' *(S05)

There was a sense that in some cases, the impact on team working with colleagues on the labour ward resulted from perceived delays in referring women from the Birth Centre who needed obstetric care, with one obstetrician reporting:

*'When women need to be transferred it becomes quite high risk. They may have been in labour for quite some time, need pain relief or there's meconium or whatever. I am aware that there have been a couple of referrals which have been rather late. The women have been in labour for a long time and were in the first stage for 12 hours or so. I'm sure it deviated from the pathway but why it happened I'm not sure and we'll be looking into it' *(S04)

The pressure of work in other areas of the unit, particularly the labour ward, also impacted on the team working of the Birth Centre midwives and their ability to provide the level of care they felt was appropriate to support normal birth:

*'we're not in a position to be able to offer the skills I've wanted because very often we have two midwives up here and if it's a quiet shift, they will take the more experienced midwife to labour ward and leave the least experienced up here.....it leaves a very vulnerable, newly qualified midwife up here in a situation where she feels isolated and on her own. Labour ward is still seen as a priority' *(S03)

### Impact on women's experiences

None of the women interviewed were aware that their care had been informed by use of a pathway although it was clear from the observations conducted that all of these women *experienced *care informed by a pathway because it was the only documentation available to record their labour progress, with each woman's labour and birth notes comprising the pathway. When asked whether they were aware of the pathway, one woman replied:

*'No, not really, had no idea. I don't remember seeing any paperwork at all. The midwives always explained what was happening and it made it clear they would have to keep checking the baby's heart rate and if I was OK' *(W03)

Another woman who had given birth to her second baby also could not recall seeing the pathway in use but felt that this may have been due to the impact of labour on her ability to take note of what was happening around her:

*No, I don't think so. I didn't remember anything like this with my first baby. Not really, to be honest, I think I was so out of it! The midwife told me all the way through what was happening and when she listened to the baby's heart beat, she always said if it was OK' *(W01)

That the midwife did inform the woman and her partner of what she was doing and why were noted during the period of observation. Interviews with the women also highlighted areas not accounted for in the pathway. One woman whose progress in early labour had been assessed several times at the unit said:

*'It wasn't made clear to me at any point that contractions could be so painful and this meant you would lose out on sleep. So I ended up really worried that I would be very exhausted'*. (W03)

The same woman was also upset at what she viewed as a lack of regular vaginal examinations, to keep her informed of her progress in labour. She later was transferred to labour ward for an instrumental (forceps) delivery:

*'I would have asked for more internal examinations....I was pushing for over an hour and a half, which I thought was too long. My waters hadn't broken, and a more thorough examination at more frequent intervals could have made a difference as to whether I would or would not be able to push the baby out' *(W03)

The midwife observed in practice caring for this woman thought the pathway did not take into account how tired a woman could become during her labour, especially if she was having her first baby:

*'The pathway didn't take account of how tired women can become. XXX had been up all night prior to coming to the Centre in labour and it was clear she was so exhausted... these women do so much. If you know a woman is so tired, it's unlikely that she'll be able to push, what do you do? As soon as XXX was transferred to labour ward, she had an epidural' *(M04)

### Changes to improve the pathway

The midwives working on the Birth Centre were asked to reflect on what changes, If any, they considered could improve the pathway. One clear issue was need to ensure that women and their partners were involved in more discussion around expectations of the progress of labour and how pain relief options were managed on the Birth Centre:

*'One problem though is that you often feel that families who come in with a woman really don't know what to expect. I had a case recently where the partner was very aggressive when we had made a decision with one woman. He'd wanted her to be transferred to main labour ward to have her labour augmented, but the woman didn't want to go at the time. She was eventually transferred after she'd had pethidine, which her partner felt had been denied by us for too long. Once her labour was over though, her came up and thanked me for going through why she didn't need to be transferred straight away and then why she was transferred' *(M04)

There were some feelings as well that slow progress of labour was an issue that needed to be further reflected on with respect to the pathway and how this was discussed with a woman and her partner:

*'I think it could provide some reminders about communication, but in reality it's so individual and so reliant on events at the time. I do think we could have a section which deals with 'slow progress' in more detail as an indication for intervention. We just can't say exactly when a baby will be born, but it's important that this is clearly discussed with the woman and her partner' *(M04)

### Impact on the organisation

The introduction of the pathway did not appear to have made any impact on use of unit resources, including postnatal stay. However as the Birth Centre had only been opened relatively recently, there was a view that it was too early to assess if the pathway had supported more effective use of resources. The difficulty in capturing the impact of the type of care women experienced on the Birth Centre as opposed to care on the labour ward was also raised:

*'I would like to think its' had an impact on unit costs. However, we deliver about 50 women a month, which means 50 less births on the main labour ward. So it's hard to say in a sense, how do you quote the women who have delivered here and what type of care they would have received on the labour ward. How many of them would have had an epidural, how many an instrumental delivery? You can't really quantify that' *(S03)

When asked if the postnatal stay on the Birth Centre had been influenced following introduction of the pathway for normal birth, there did not appear to have been any reduction as early discharge remained a unit wide policy:

*'in the leaflet we say it is a 6 - 24 hour stay because you are low risk and all of that. We've never, I shouldn't say never, may be once a fortnight or once every 3 weeks on the postnatal bay, we have the scope to say 'would you like to stay another night?' *(S03)

There was a positive view that the pathway had led to a reduction in the duplication of care particularly with respect to duplication of written information:

*'Without a doubt. No note keeping. There are no written notes apart from filling in the pathway. So that helps' *(S03)

## Discussion

This study examined the impact of the introduction of a pathway to support normal labour and birth in one English birth centre on midwives, women and other key stakeholders within the organisation. As far as we are aware, this is the first time that the outcomes of an adapted version of the AWP has been evaluated in an English birth setting, despite anecdotal evidence of introduction into units across England. Findings showed a wide range of views as to the impact of the pathway on midwifery practice, decision making and multi-professional team relationships and highlighted a lack of awareness among women of how their care was guided and informed. Although data are based on one case study, with several quotes selected to reflect identified themes representing the views of one particularly insightful interviewee, there are a number of important issues which need to be considered if pathways to standardise decision making in labour and birth care are to be used. The study findings emphasise that implementation of this complex intervention can include achievement of anticipated benefits but can also include unintended consequences.

Despite the wide promotion of care pathways within the NHS, the lack of evaluation is noticeable. The original AWP, which was developed for midwives in an attempt to increase normal birth and inform a continuum of care from the latent phase of labour to completion of the third stage was rolled-out during 2002 - 2004 to every maternity unit in Wales with no accompanying evaluation strategy [22]. Although grading of the levels of evidence used to support the content of the pathway is included in the original AWP, the supporting references are not cited. The adapted version evaluated in the current study did not include any supporting evidence within the documentation and no mention was made during the interviews of the support of the pathway to promote evidence based practice - the focus was support for midwifery practice.

A decision was taken by midwives in the current study to not include the original Part 1 (telephone consultation) in the adapted AWP after piloting because it was considered a 'paper exercise'. There were concerns about how confidential information reported during a telephone call would be recorded and how to ensure the correct paperwork was placed into the correct notes. Spiby and colleagues [23] published findings of an exploratory study of the use of Part 1 of the AWP as part of a larger study to investigate care for women in early labour (the OPAL study). Focus groups with midwives at two maternity units in Wales highlighted a number of issues including training, difficulties encountered when changing from traditional documentation to a pathway, potential for duplication of information if women contacted the unit more than once and for midwives to not exercise professional judgement. Midwives did report that use of Part 1 saved time and could inform workload planning [23]. Interviews with a sample of women whose care had been informed by Part 1 of the AWP (n = 46) found they were satisfied with choices offered and being treated as an individual. They were dissatisfied with unclear instructions, confusion over when to go to hospital, lack of support, reassurance and continuity of care if they later spoke to another midwife. Few women reported that use of the pathway had been discussed with them.

The other major adaptation of the AWP in the current study was that maternal and fetal assessments during the latent phase of labour were undertaken every hour, rather than every 30 minutes. This decision was based on the fact that women had low risk pregnancies; instead of being at home and awaiting onset of established labour (where no routine observations would be undertaken), they were on the Birth Centre. The hour decision was a compromise as some midwives did not consider observations were necessary, whereas others were concerned that the woman should be assessed as she had been admitted to the Birth Centre. This amendment appeared to be a pragmatic decision which reflected midwifery experience and views. NICE [24] recommends women in suspected labour have the fetal heart auscultated for one minute after a contraction.

When work to introduce and adapt the pathway commenced, the obstetricians interviewed were of the opinion that the pathway was a 'midwifery' thing. This in turn was viewed by the midwives responsible for its introduction as an appropriate response. On reflection, midwifery key stakeholders felt that if active engagement of their obstetric and other midwifery colleagues had been sought at the outset, it may have reduced some of the later tensions and concerns the Birth Centre midwives had to deal with. In a recent qualitative evaluation of the impact of the introduction of the AWP in two maternity units in Wales by Hunter [22], similar issues with respect to the lack of engagement of the maternity team were reported, however unlike Hunter's study [22], implementation of the adapted AWP only took place in one part of the study site. This raises several issues - how to share a philosophy of normal labour and birth with colleagues who do not routinely care for low risk women, and acknowledgement that a woman's care, even if low risk at the onset of labour, may later necessitate transfer to the main labour ward and input of the multi-professional team. That the adapted AWP was viewed by the obstetricians as a 'midwifery' thing was similarly reported at the other four corresponding clinical sites where this study took place [9]. Protocols and pathways were viewed by the medical staff in the other study sites as a 'nursing' thing, a perspective even reported at sites where a standardised care approach was intended for use by the multi-professional team. As Rycroft-Malone et al. [9,13] suggest because standardised care tools such as care pathways, protocols and checklists are perceived differently by individuals and professions, they are socially and professionally constructed phenomena. If this is the case, the goal of standardisation is unlikely to be achieved because use of these tools will be mediated by individual and professional factors, as recently highlighted by Bosk and colleagues with respect to use of checklists in medicine [25]. It is also problematic that whilst tools such as care pathways promote a linear approach to a patient journey, human behaviour and countless other complex factors which influence decision making along the way may not result in the outcome of care anticipated [26].

The introduction of the pathway was not viewed by the Birth Centre midwives as a substitute for clinical judgement. During the observation periods it was noted that the pathway was completed in retrospect, for example, following maternal and fetal observations the midwife would leave the room to complete the pathway. This may have had more to do with the physical space available in the birth rooms which did not include a table or other space for the midwife to use but it did limit collation of data on the extent to which the pathway informed 'next steps' in a woman's labour progress. Whilst the midwives welcomed the support the pathway offered their practice, some encountered difficulties adjusting to the minimal level of documentation required - the original AWP refers to 'documentation by exception'. The premise is that as the woman's care is 'normal', the partogram (on which the progress of labour is plotted) is the main form of record keeping. In addition to practical difficulties reported, the lack of documentation is also likely to lead to other unintended consequences. Hunter [22] highlighted in her study that the use of the pathway could result in the loss of the woman's 'story' as the narrative of her care and experiences are not captured. This absence of information may have implications for aspects of maternal well-being and postnatal recovery, as women who could benefit from discussion of their birth experiences will have gaps in their labour and birth history which could be difficult to resolve. The absence of documentation may also be problematic with respect to informing future pregnancy plans and management. Further research and evaluation on the impact of streamlining labour records (such that the normal birth pathway encourages) is required.

The lack of documentation was also raised as a potential litigation issue, which Hunter [22] also reported. In contrast to Hunter's study [22] where midwives raised these concerns, the obstetricians in the current study mooted this. When a woman was transferred to their care, they were unable to gauge from the pathway that the Birth Centre midwife had done the 'right thing at the right time'. As a consequence, Birth Centre midwives felt they had to constantly defend their practice and management of a woman's care with their obstetric colleagues. The health of women in the UK who become pregnant is increasing in complexity [27,28]. Recent high profile maternity service investigations have highlighted poor communication and working relationships between midwives and obstetricians [29] indicating that the maternity services have to address how joint working relationships can be enhanced if new technology is introduced to support practice and clinical care in one sector of the service. Although obstetric and midwifery professional boundaries and spheres of practice are clear, the health needs and care requirements of women during pregnancy and labour are not always easy to predict and can alter rapidly. In addition, if there is no clearly linked evidence base presented within a pathway, those using it will need to ensure familiarity with the evidence and ability to apply this as appropriate in each individual case.

It is also clear that a pathway per se is not the only factor that affects a woman's care or her birth outcomes which the Birth Centre midwives were keen to stress; the environment of birth, the philosophy of care and the skills and expertise of the individual midwife were all viewed as important. The role of the Birth Centre manager was also raised by some of the midwives interviewed as important to support their practice and role in normal birth. Nevertheless, for the obstetrician's at the study site, the pathway was a cause of concern because it provided insufficient documentation that a midwife had appropriately managed the labour of a woman prior to her transfer to their care.

Few studies have addressed the extent to which litigation drives the content of maternity care and decisions about labour and birth management including transfer of care. Hindley and Thomson [30] explored midwives views around the use of routine electronic fetal monitoring for women whose pregnancies were low risk, an intervention unsupported by evidence [24] but viewed by the midwives in their study as a valuable form of evidence if litigation was instigated. The rise in the caesarean section rate has been viewed as a form of defensive practice in a number of developed countries including the UK and the USA [31,32]. The litigation pressures on UK maternity staff should not be underestimated - obstetrics and gynaecology accounted for 21% of cases reported to the NHS Litigation Authority (NHSLA) and 51% of the total value of claims paid from April 1995 to March 2008 [33]. Work around how services revise and review litigation pressures on maternity care staff is urgently needed if multi-professional working, confidence with respective roles and support for normal labour and birth are to be promoted in line with current maternity service policy [3].

The pathway was perceived as having a positive impact on the midwives autonomy and confidence in their skills, particularly for newly qualified midwives. Midwives qualified for longer expressed the view that the pathway supported how they would practise anyway. The sense that the pathway was one of several factors that influenced normal birth practice was emphasised, thus it was unclear if the pathway per se supported autonomy or was a contributing factor. One key stakeholder reported a sense that some of the midwives who did not work on the Birth Centre felt the pathway had a detrimental impact on autonomy because it 'told midwives what to do'. From the interviews, the Birth Centre midwives viewed the pathway as a support for their practice - they would continue to make decisions based on their clinical judgment and not solely informed by the pathway. However, if pathways are to be more extensively used the issue of how use impacts on clinical decision making and clinical judgement has to be more fully explored, especially if pathway documentation do not include a written narrative of care.

Another positive consequence of use of the pathway was the perceived impact on the team working of the Birth Centre midwives, which was in contrast to the views of the impact on relationships with colleagues elsewhere in the unit. Use of the pathway promoted communication sharing as a midwife taking over the care of a woman could see immediately how her care was progressing. Hughes et al. [34] undertook a series of focus groups with midwives to improve the understanding of factors affecting midwifery morale and how to enhance engagement of midwives in key strategic service planning and service re-development initiatives. Poor midwifery working relationships were influenced by a lack of support for one another and the influence of working in a 'culture of blame'. Following management and other organisational changes at the same unit, another series of focus groups highlighted that changes to encourage cultural shifts in midwifery practice could have a positive influence on working relationships. It is likely that the establishment of the midwifery-led Birth Centre in the current study was a catalyst for enabling midwives to work within an environment which fully supported their skills and philosophy and positively impacted on their working relationships.

One limitation of the study is the small sample size. It was apparent that in some cases, the midwives caring for a recruited woman did not contact the research team to inform them of her admission in labour, particularly if this was late at night or the early hours of the morning. In other cases, it was difficult for a member of the research team to get to the Birth Centre to complete an observation due to other work commitments as observation periods could not be planned for obvious reasons. Nevertheless, obtaining the views of the women who were recipients of care informed by the pathway was an important component of this study and we have highlighted some important issues. In line with the other clinical areas included in the overall project [9], none of the six women interviewed were aware that a pathway had informed their care. Hunter [22] and Spiby et al. [23] also reported that the women in their studies were frequently unaware of the use of the All Wales pathway. The current study highlights a potential 'gap' between women's and midwives views of normal labour and birth. The women interviewed were clear that they wanted to give birth on the Birth Centre but in terms of labour progress would see 'how it goes', whereas the midwifery perspective was one of support to enable a woman to maintain progress within the pathway to achieve normal birth. One woman spoke of her concern that she was unaware that labour could take time to become established or that contractions experienced during this phase could be painful. She had been admitted and discharged in latent labour three times prior to the onset of established labour and spoke of her tiredness when labour was finally confirmed. She felt that more frequent vaginal examinations could have informed an earlier decision to transfer her to the labour ward and she remained unhappy about this aspect of her care. From the pathway perspective, as she was 'normal' and there were no indications otherwise, there would have been no prompt to intervene earlier. Although the other women interviewed were positive about their care, the importance of effective communication and sharing of the philosophy of care to support normal birth cannot be underestimated. A core theme of current maternity service policy is that women are informed and engaged in all aspects of decision making [3]. The impression from this study was that whilst women were advised of what was happening at set points during their labour, their input to decisions and the role of the overall framework informing why interventions would or would not be undertaken was not sufficiently emphasised.

## Conclusion

Study findings add to growing evidence that the introduction of a care pathway to support normal labour and birth can result in a number of anticipated and unintended consequences. Whilst there were undoubted benefits from the perspective of the skills, practice and autonomy of the Birth Centre midwives, issues related to the negative impact on collaborative multi-professional team working need to be explored more fully particularly given current concerns about the safety and quality of maternity care in the UK. The 'language' of normal labour and birth needs to be unpicked and explored from the perspective of the midwife, the obstetrician and the woman as the differing views identified here are not conducive to effective communication and highlight the need for clarity and greater understanding. The commonly held view that care pathways are useful tools to support standardised decision leading to the delivery of care which is appropriate, timely and informed by evidence needs to be revisited as does the salutary lack of robust evaluation to accompany the introduction of pathways into practice.

## Competing interests

We declare we have no competing interests and were not in clinical practice at the study site.

## Authors' contributions

The original idea for the study was proposed by JRM. JRM was the lead investigator for the SDO funded project, DB was a co- investigator. DB wrote the paper and JRM and MF contributed to it. All authors approved the writing of the text.

## Pre-publication history

The pre-publication history for this paper can be accessed here:


